# Effect of heat inactivation and bulk lysis on real-time reverse transcription PCR detection of the SARS-COV-2: an experimental study

**DOI:** 10.1186/s13104-022-06184-z

**Published:** 2022-09-07

**Authors:** Dereje Leta, Gadissa Gutema, Gebremedhin Gebremichael Hagos, Regasa Diriba, Gutema Bulti, Tolawak Sura, Desta Ayana, Dawit Chala, Boki Lenjiso, Jaleta Bulti, Saro Abdella, Habteyes Hailu Tola

**Affiliations:** 1 HIV/AIDS Disease Research Team, TB and HIV/AIDS Disease Research Directorate, Ethiopian PublicHealth Institute, Addis Ababa, Ethiopia; 2 Department of Medical Laboratory Sciences, College of Health Sciences, AddisAbaba University, Addis Ababa, Ethiopia; 3 TB Disease Research Team, TB and HIV/AIDS Disease Research Directorate, EthiopianPublic HealthInstitute, Addis Ababa, Ethiopia

**Keywords:** Heat inactivation, SARS-COV-2, RT- PCR, Ct value

## Abstract

**Objective:**

This study aimed to investigate the effect of heat inactivation and chemical bulklysis on SARS-CoV-2 detection.

**Results:**

About 6.2% (5/80) of samples were changed to negative results in heat inactivation at 60 °C and about 8.7% (7/80) of samples were changed to negative in heat inactivation at 100 °C. The Ct values of heat-inactivated samples (at 60 °C, at 100 °C, and bulk lysis) were significantly different from the temperature at 56 °C. The effect of heat on Ct value should be considered when interpreting diagnostic PCR results from clinical samples which could have an initial low virus concentration. The efficacy of heat-inactivation varies greatly depending on temperature and duration. Local validation of heat-inactivation and its effects is therefore essential for molecular testing.

## Introduction

### Background

Coronavirus disease 2019 (COVID-19) is a newly emerged human infectious disease caused by severe acute respiratory syndrome coronavirus-2 (SARS-CoV-2) (1). SARS-COV-2 is a single-stranded Ribonucleic acid (ssRNA). Based on the rapid rate of increase in humans, the World Health Organization (WHO) classified the COVID-19 outbreak as a pandemic by the end of 2019 [1–3]. Since it reached a critical point in March 2020, WHO declared that the world needs speedy and quick solutions to diagnose and tackle the further spread of COVID-19 [4, 5]. The nucleocapsid region and the open reading frame (ORF)-1 of SARS-COV-2 are the most ideal amplification target [6, 7].

The CDC rRT-PCR panel for detection of SARS-CoV-2 demonstrated high sensitivity and specificity for detecting RNA with no observed false-positive reaction [8]. These assays have proven to be valuable for rapid laboratory diagnosis and control of COVID-19 [9, 10]. Laboratory viral nucleic acid (NA) testing using RT-PCR assays is currently the “gold standard” for the diagnosis of COVID-19 [1, 4, 11, 12].

Buffer-based NA extraction methods to obtain high-quality NA has not been developed primarily for the inactivation of infectious samples [13, 14]. Automated NA extraction is often performed outside of the biosafety cabinet. To avoid aerosol formation, a pre-inactivation step under appropriate biosafety conditions is an absolute requirement [13–16].

Accordingly, the extraction of viral RNA requires the first step of lysis or heat inactivation of the virus at different temperatures and minutes [7, 17]. In our laboratory at the Ethiopian Public Health Institute (EPHI), we usually do heat inactivation for 30 min at 56 °C. The impact of a higher temperature on SARS-COV-2 detection has not been thoroughly examined [17, 18]. Still, there is a lack of understanding of the molecular-level changes that are taking place in the virus due to the different heat and chemical conditions [19, 20].

The effect of heating at different temperatures and time periods prior to testing remains unclear (still under investigation). Thus, this study aims to evaluate the effect of heat inactivation at different temperatures and times and to determine the effect of chemical inactivation by bulk lysis on SARS-CoV-2 detection.

## Main text

### Materials and methods

#### Study area and design

The laboratory-based experimental study design was conducted at EPHI National HIV Reference laboratory from August to November 2020. The laboratory has a well-established quality system and is ISO 15189; 2012 accredited for HIV Viral load testing and early infant diagnosis.

#### Sample size determination and sampling method

Eighty Nasopharyngeal/Oropharyngeal swab samples were selected and taken out from -80^0^C storage. Positive samples with known threshold cycle (CT) value were selected by simple random sampling technique, from 9,520 positive samples within one month, from 28.4% prevalence of Covid-19 in Ethiopia as of August 20/2020. About 2380 samples were done in one week, then dividing this sample by 80 is 30, which was the interval number by which samples were selected. The first sample was selected by the lottery method.

#### Specimen collection and testing

**Fig. 1:**
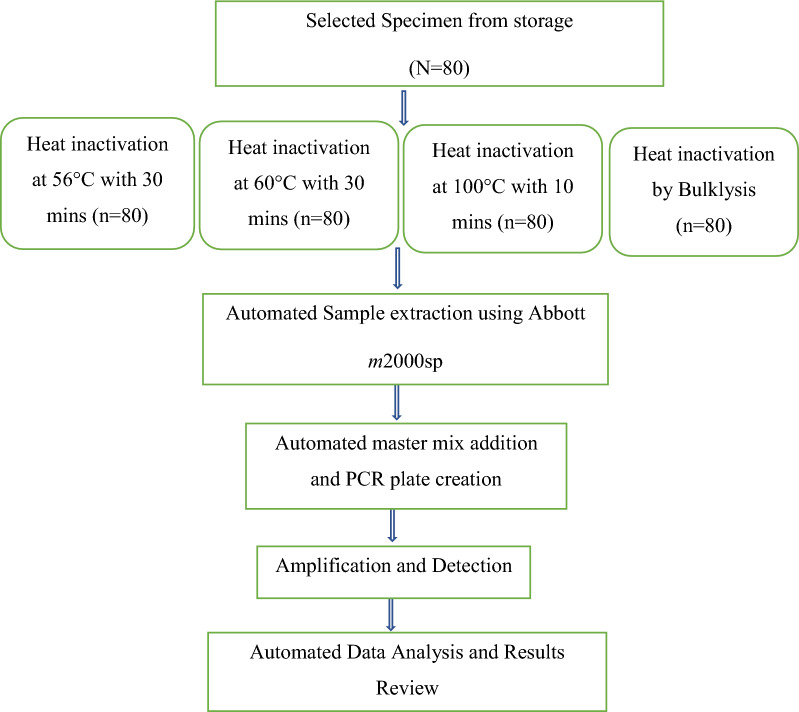
Process of specimen testing

All samples were tested with 2 controls (1 positive, 1 negative) when testing by reference method (Abbott Real-Time SARS-COV-2(EUA)). Figure 1 Depicts the overall experimental procedure followed.

**Fig. 2:**
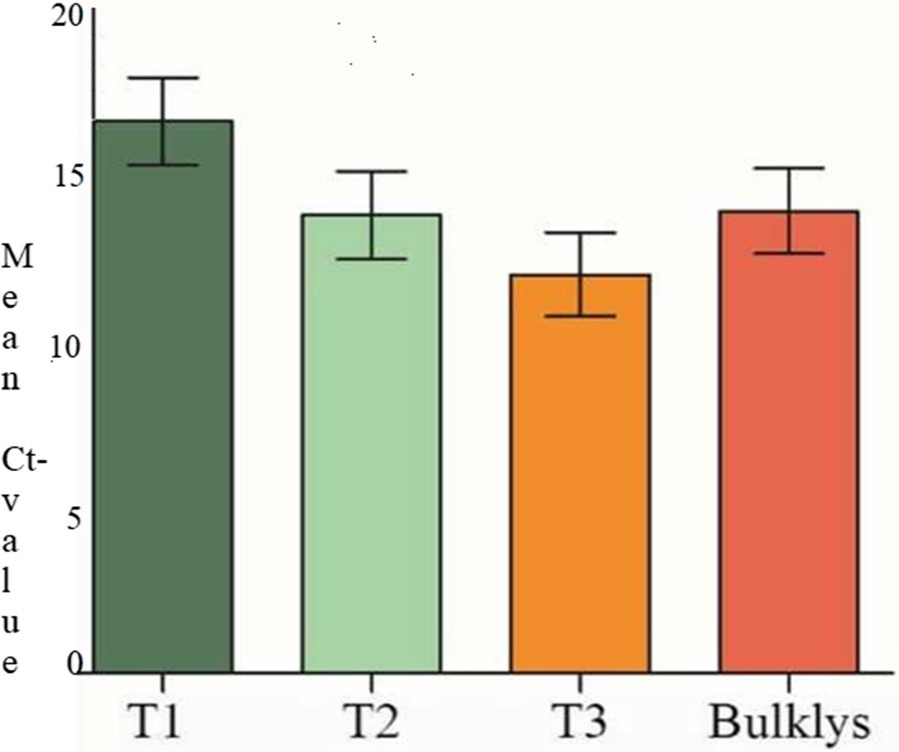
Mean cyclic threshold comparison between the three temperatures and bulklysis. (The standard errors bar shows mean ± SD): T1-Temperature1 = at 56 °C; T2-temperature 2 = at 60 °C and T3- temperature 3 = at 100 °C; *SD *standard deviation)

### Experiment

Tests were performed using the Abbott Real-time SARS-CoV-2 assay, a rRT-PCR test for the qualitative detection of SARS-CoV-2 in the samples [21]. A dual target assay for RNA-dependent RNA polymerase (RdRp) and N-genes detection of NAs from SARS-CoV-2 was analyzed. Results were reported as positive if the Ct value was < 32, and defined as negative if the Ct value was 32 or more, based upon the manufacturer [1].

The effects of heat treatment at different temperatures and durations and chemical inactivation on the SARS-CoV-2 rRT-PCR Ct-value were evaluated. The samples were inactivated at different temperatures and minutes in a water bath (at 56 °C for 30 min (n = 80), at 60 °C for 30 min (n = 80), and at 100 °C for 10 min (n = 80)) and by chemical bulklysis (n = 80). WHO recommends heat inactivation at 56 °C for 30 min and Abbott real-time RT-PCR as the golden method for SARS-COV-2 detection [22–24].

After viral heat inactivation, NA extraction was done from a 0.6 ml sample volume on the Abbott *m*2000*SP* instrument by using the Abbott *m*Sample Preparation System. On the other hand, Chemical inactivation was performed to see the effect of bulk lysis on the SARS-CoV-2 rRT-PCR. For chemical inactivation, bulk lysis was tested with the appropriate composition of sample and lysis proportion. Each sample was incubated with the bulk lysis buffer at room temperature for 30 min; then, the sample was extracted.

After the extraction was completed, the samples of heat and chemical treated group was detected by Abbott m2000*rt* (12). The viral RNA was extracted from 500 μL of each sample, and the final elute was 200 μL by using elution buffer [1, 22]. The SARS-CoV-2 and IC-specific probes were each labeled with a different fluorophore (FAM™ (Carboxyfluorescein), ROX™, (Carboxy-X-rhodamine), and VIC® P (Proprietary dye) for target and IC detection [1].

### Quality assurance

All laboratory procedures were performed as per the documented SOP as manufacturing recommendations.

### Data analysis

Data was analyzed and described by mean and standard deviation (mean ± SD). Normality was assessed for the three temperature scenarios and bulk lysis, and they were violated normality. However, after we transformed by natural logarithm all of them were normally distributed. Repeated measurement analysis of variance was used to assess the mean difference between temperatures and bulk lysis. To identify the place of significant difference post hoc analysis with modified Bonferroni correction was used.

### Results

#### Reverse transcriptase real-time polymerase chain reaction results of heat-inactivated samples at different temperatures and durations

The effect of heat treatment at different temperatures and durations on the SARS-CoV-2 rRT-PCR Ct-value was evaluated. All heat-inactivated samples at 56 °C for 30 min were tested positive. The Ct values of RdRp were 4.37–31.03 CN (cyclic number) at 56 °C for 30 min, 3.68–30.64 CN at 60 °C for 30 min, 3.37–28.74 CN at 100 °C for 10 min. Chemical bulklysis inactivated samples were from 3.62 to 27.74 CN except for those with weak positive samples (Table 1) which were turned to negative results, as compared to the heat-inactivated samples at 56 °C. Heat inactivation methods resulted in the reduction of positive SARS-CoV-2 samples to undetectable levels, especially in weak positive samples. About 6.2% (5/80) and 8.7% (7/80) of samples were changed to negative results in heat inactivation at 60 °C (30 min) and 100 °C (10 min) respectively. A comparison of heat inactivation of weakly positive samples at 56 °C with (at 60 °C, 100 °C, and chemical bulklysis) was summarized (Table 1).

**Table 1:** Cycle threshold values for RdRp measured from swab samples following heat treatments at different temperatures and chemical bulklysis

S.N	Ct values results			
	T1	T2	T3	Bulklysis
1	31.03 CN	Negative	Negative	Negative
2	30.11 CN	Negative	Negative	Negative
3	28.57 CN	Negative	Negative	Negative
4	27.13 CN	28.37 CN	Negative	27.74 CN
5	26.22 CN	30.64 CN	Negative	26.62 CN
6	29.65 CN	Negative	Negative	28.89 CN
7	30.54 CN	Negative	Negative	28.99 CN
%		62%	8.7%	3.7%

*T1* temperature at 56 °C, *T2* temperature at 60 °C, *T3* temperature at 100 °C, *CN* Cyclic number, *Ct* Cyclic threshold

The Ct values of heat-inactivated samples at (60 °C, 100 °C) were significantly different from the temperature at 56 °C (as compared to 56 °C) (*p* = 0.01, *p* = 0.001) respectively. The place of significant difference was identified by using post hoc analysis with modified Bonferroni correction (Table 2).

**Table 2:** Mean difference of group with their confidence interval and *p*-value following heat inactivation at different temperatures and durations EPHI, Ethiopia

Group	Groups	MD (95%CI)	*P*-value
T1	T1 vs T2	0.10 (0.02 to 0.18)	0.01
	T1 vs T3	0.15 (0.10 to 0.24)	0.001
	T1 vs Bulk lysis	0.13 (0.02 to 0.24)	0.02
T2	T2 vs T1	− 0.1 (− 0.20 to − 0.02)	0.01
	T2 vs T3	0.05 (− 0.01 to 0.11)	0.16
	T2 vs Bulk lysis	0.03 (− 0.01 to 0.13)	0.999
T3	T3 vs T1	− 0.15 (− 0.24 to − 0.1)	0.001
	T3 vs T2	− 0.05 (− 0.11 to 0.01)	0.16
	T3 vs Bulk lysis	− 0.02 (− 0.12 to 0.10)	0.999
Bulk lysis	T1	− 0.13 (− 0.24 to − 0.20)	0.02
	T2	− 0.03 (− 0.13 to 0.10)	0.999
	T3	0.02 (− 0.10 to 0.12)	0.999

*MD* mean in difference, *CI* Confidence interval, *T1* temperature at 56 °C, *T2* temperature at 60 °C, *T3* temperature at 100 °C

#### Heat inactivation by chemical bulk lysis

All samples were incubated with chemical bulk lysis buffer, (as it was compared with AVL buffer, AVL serves as the standard for the comparison of the diverse chemical inactivation methods). The decline in the viral RNA quantity was observed in some of the samples treated with chemical bulklysis, especially for those with high Ct values. It was shown that 3.7% (3/80) of analyzed samples were turned to negative. There were significant differences between the Ct values of bulklysis and heat inactivation at 56 °C for the RdRp genes (Repeated measure ANOVA; *P* = 0.02). Following the comparison of bulk lysis to all other forms of inactivation used in this study, only heat-inactivated at 56 °C was significantly different from bulklysis inactivation (Fig. 2).

### Discussion

The SARS-CoV-2 disease has as of late risen and quickly spread in people causing a critical danger to universal wellbeing and the economy.

Respiratory specimens have been used to diagnose SARS-CoV-2 infection by Abbott rRT-PCR, and are regarded as the main detection method. Following the rapid global spread of SARS-CoV-2 and the need for universal testing, more and more individuals are exposed to non-inactivated virus samples. The WHO and United States CDC have released laboratory guidelines to mitigate the risk of exposure during diagnostic and research procedures [25–27]. The proceeded to require COVID-19 testing worldwide requires the utilization of straightforward and viable inactivation techniques.

It has appeared that within the SARS-COV-2 swab test, the amount of SARS-COV-2 might be decreased at 60 °C for 30 min and in bulklysis, but still irresistible. As it was heating at a temperature of 100 °C for 10 min was able to inactivate it [12].

However, we have been trying to demonstrate that it is possible to ensure the test integrity by applying heat inactivation under several conditions. RT-PCR Ct values are defined as the number of cycles of amplification required for the accumulated fluorescence (produced by target gene amplification) and are inversely related; low Ct values indicate high viral loads and high Ct values indicate low virus NA concentration in the sample [28]. In this study, the Ct value was essentially influenced by warming at 60 °C and 100 °C for those with weak positive samples. This is in agreement with the studies of Pastorino [12]. The less eminent increase in Ct value observed when the virus was heated to 100 °C, can be ascribed to the shorter warming time, this can be in line with the study by Zou et al. [26]. Lower temperature heat treatment combined with chemical inactivation, short-duration high-temperature heat treatments, or chemical inactivation alone may be more suitable to protect RNA integrity and maximize PCR optimization for the discovery of SARS-COV-2 RNA from low-concentration SARS-COV-2 samples. Our results show significant variation in the effect of heat-treatment inactivation on the SARS-CoV-2 detection. This emphasizes the significance of local approval of inactivation strategies and the need for consistency in inactivation protocols.

Weak positive samples may become false negatives in SARS-CoV-2 RT-PCR detection. Our study also has shown that the heat-inactivated samples at 56 °C were consistent with those in heat-inactivated ones at 60 °C, 100 °C, and chemical bulk lysis for low Ct value results, which agrees with the study done by Pastorino, Rao, and Pan [12, 29, 30].

Warming at 100℃ for 10 min would result in untrue negative, which is steady with that of warming at 92℃ for 15 min, the SARS-CoV-2 RNA in a test was dropped altogether as the study done by Zou et al. and Burton [26, 31]. In reality, considers has been proposed that the test cells should pass on and burst within the occasion of moderately high temperature and long terms, driving to the discharge high number of cell nucleases, and after that, a huge sum of RNA debasement, which may contribute to untrue negative in NA detection. We hypothesize that heating at 100℃ for a long period of time lyse a huge number of cells, and leaves out RNA to RNases enzyme shown within the tests.

Although our study showed that heating at 100 ℃ for 10 min was steady with heat at 56 °C, except for those tests with frail positive, strong positive samples appeared an inclination to diminish in Ct values to a few extents, this is in agreement with the study done by Wang [32]. The RNA conservation may be due to the conservation chemical, which contains guanidine isothiocyanate. This proposed that the nearness of the conservation chemical can viably ensure the keenness of the viral NA, in this manner expanding the extent of recognizable NAs. However, numerous components can impact the effect of the lysis buffer, the amount of virus, nature of the network, contact time, and response temperature, concentration/composition of the lysis buffer used.

### Conclusion

We found that the effect of heat-inactivation varies greatly depending on temperature and duration. The impact of chosen inactivation method on the sensitivity of subsequent SARS-CoV-2 detection should be assessed locally. The effect of heat on Ct value should be considered when interpreting diagnostic PCR results.

### Limitation

Use of the Abbott Real-time SARS-CoV-2 assay is limited to trained personnel.

Performance has only been established with the specimen types listed in the Intended Use. Other specimen types have not been used with this assay.

## Data Availability

The original contributions presented in the study are included in this published article /supplementary material. Further inquiries can be directed to the corresponding author.

## Abbreviations

CDC: Center Disease Control and Prevention
CT: Cyclic threshold
CN: Cyclic number
EPHI: Ethiopian Public Health Institute
EUA: Emergency Use Authorization
IC: Internal control
ISO: International Standard for Organization
SARS-COV-2: Severe Acute Respiratory Syndrome Coronavirus 2
SOP: Standard Operating Procedures
RNA: Ribonucleic acid
WHO: World Health Organization
RT-PCR: Real-time polymerase chain reaction
RdRp: RNA dependent RNA polymerase
VTM: Viral Transport Media

## Declarations

### Facilities available for the study

The study was conducted in the Ethiopian public health institute. We were also, using suitable data storage devices and a recent version of statistical packages that are available at EPHI for data storage and analysis.
